# A pre-trained language model-based cross-modal fusion framework for predicting miRNA-drug resistance and sensitivity associations

**DOI:** 10.1371/journal.pcbi.1013968

**Published:** 2026-02-10

**Authors:** Nan Sheng, Yunzhi Liu, Ling Gao, Wenju Hou, Lan Huang, Yan Wang

**Affiliations:** 1 College of Computer Science and Technology, Jilin University, Changchun, China; 2 Key Laboratory of Symbolic Computation and Knowledge Engineering of the Ministry of Education, Jilin University, Changchun, China; Guangxi University, CHINA

## Abstract

MicroRNAs (miRNAs) are pivotal regulators of drug resistance and sensitivity in cancer cells, functioning as tumor suppressors or oncogenes that modulate the cellular response to anticancer drugs. While experimental identification of miRNA-mediated drug resistance and sensitivity is both costly and laborious, computational methods present a promising alternative. Recent advances in pre-trained language models (PLMs) offer new opportunities to leverage large-scale unlabeled biomolecular data for enhanced relationship prediction. In this study, we introduce PLMF-MDA, a PLM-based cross-modal fusion model designed to predict miRNA-drug resistance (MDR) and miRNA-drug sensitivity (MDS) associations. PLMF-MDA integrates miRNA and drug multimodal embeddings derived from PLMs and intrinsic feature extractors, and employs a cross-modal attention fusion module to adaptively capture key interactions between modalities. To evaluate the performance of the approach, we manually constructed two benchmark datasets. Experimental results demonstrate that the PLMF-MDA achieves superior prediction performance. Furthermore, case studies on anticancer drug docetaxel and gefitinib demonstrate its potential in discovering novel MDR (MDS) associations. All data and source code are available on GitHub: https://github.com/sheng-n/PLMF-MDA.

## Author summary

MicroRNAs (miRNAs) are important modulators of cancer cell response to chemotherapy, but experimentally identifying miRNA-mediated drug resistance and sensitivity relationships is slow and resource-intensive. Here we present PLMF-MDA, a computational framework that leverages pre-trained language models (RNA-FM and ChemBERTa-2) to generate global embeddings for miRNA sequences and drug SMILES, and combines these with fine-grained nucleotide- and atom-level representations learned by multi-scale CNN and GCN. A cross-modal attention fusion module adaptively integrates these heterogeneous features to capture key interactions between miRNAs and drugs. We curated two benchmark datasets (MDRdataset and MDSdataset) to evaluate the approach, PLMF-MDA consistently outperforms existing methods on AUC and AUPR metrics and maintains strong performance on datasets containing previously unseen nodes. A case study on the anticancer drug docetaxel and gefitinib further demonstrates the model’s ability to prioritize plausible novel miRNA-drug resistance and sensitivity candidates. PLMF-MDA can serve as a practical tool for researchers by identifying miRNAs that may potentiate the efficacy of specific drugs, thereby helping to narrow experimental targets and accelerate the development of more effective cancer therapies. All data and source code are freely available at to facilitate reuse and further research by the community.

## Introduction

Cancer represents the second leading cause of death globally, following cardiovascular disease, and poses a severe threat to patient life and health [1]. Currently, surgical resection, radiation therapy, targeted therapy, and chemotherapy constitute the primary treatment methods for cancer [2,3]. Among these approaches, chemotherapy serves as the first-line standard treatment protocol across all cancer stages. However, the emergence of drug resistance significantly limits the clinical application of chemotherapeutic drugs, ultimately leading to treatment failure and patient mortality. Recent research has demonstrated that miRNAs, small non-coding RNAs approximately 19-25 nucleotides (nt) in length, can participate in tumor cell drug resistance by targeting drug resistance-related genes or influencing genes associated with cell proliferation, cell cycle regulation, and apoptosis [4]. For instance, miR-301b-3p has been shown to suppress TXNIP expression, thereby promoting cisplatin and vincristine resistance in gastric cancer cells and providing novel insights for gastric cancer chemotherapy [5]. Recent evidence indicates that miR-590-5p promotes cisplatin resistance in ovarian cancer through regulating hMSH2 expression [6]. Notably, certain miRNAs also serve as potential therapeutic targets for enhancing drug sensitivity. Du et al. demonstrated that miR-375 promotes cisplatin sensitivity in lung adenocarcinoma, potentially offering new therapeutic strategies [7]. Furthermore, Wu et al. established that miR-204b enhances osimertinib sensitivity in non-small cell lung cancer by targeting CD44 to suppress cancer stemness [8]. Therefore, elucidating miRNA-mediated drug resistance and sensitivity mechanisms is crucial for rational drug development and clinical treatment strategy optimization.

Since traditional laboratory experiments for inferring these potential resistance and sensitivity relationships are both expensive and time-consuming, they present significant challenges for large-scale exploration of novel therapeutics. As an alternative, computational methods offer a promising approach to narrow down the number of potential miRNA-drug resistance and sensitivity pairs requiring investigation.

In recent years, several computational approaches have been developed to identify miRNA-related drug resistance and sensitivity associations, most of which are graph-based methods. For example, Huang et al. constructed miRNA-drug heterogeneous graphs using miRNA expression profiles, molecule graph, gene ontology, disease ontology, and known MDR associations. They employed graph convolutional neural network (GCN) to extract node features and predict drug resistance-related miRNAs [9]. Deng et al. integrated miRNA and drug similarity networks with known MDS associations to construct miRNA-drug heterogeneous graphs, proposing a dual-channel heterogeneous graph neural network model for node feature extraction [10]. Wei et al. leveraged known sensitivity relationships between miRNAs and drugs to develop a graph collaborative filtering-based contrastive learning model for inferring potential MRS associations [11]. Zheng et al. constructed non-coding RNA (ncRNA)-drug bipartite networks without considering ncRNA types and employed LightGCN for node feature extraction, using inner products to predict associations between ncRNAs and drug resistance [12]. Liu et al. integrated miRNA sequence similarity, drug SMILES similarity, and known MDS associations to build miRNA-drug heterogeneous networks, utilizing graph attention networks for node feature aggregation [13]. Recently, Sheng et al. proposed a method that leverages attribute information of miRNAs and drugs instead of commonly used interaction graph information, but it failed to distinguish between predictions of drug resistance and sensitivity [14]. Zhang et al. not only extracted miRNA and drug features from miRNA-drug bipartite networks but also employed temporal convolutional networks and bidirectional long short-term memory to learn drug fingerprint features and miRNA sequence features, respectively [15]. Sheng et al. integrated multi-source information, including miRNAs and drug-related genes, and proposed a GCN with attention mechanisms to predict miRNA-associated drugs [16]. Ouyang et al. introduced a meta-path-induced graph sparse transformer deep matrix factorization method to predict MDS associations based on the miRNA-drug bipartite graph [17].

While these methods demonstrate satisfactory performance, they typically suffer from one or two of the following limitations. (1) They generally rely on constructed miRNA-drug resistance/sensitivity heterogeneous networks, where sparsity in annotated association data affects the prediction accuracy and generalization capability of these models. (2) Graph neural network approaches based on heterogeneous graphs may not generalize well to novel drugs or miRNAs. Nevertheless, these methods represent significant advances in the MDR and MDS fields and continue to advance the possibilities of computational drug discovery.

In recent years, PLMs have profoundly impacted modern natural language processing by leveraging self-supervised learning to acquire significant representations that can be fine-tuned for various downstream tasks [18]. Given the similarity between the “language” of RNA sequences/SMILES and textual language, PLMs have been extended to RNA and molecular-related domains, such as RNA-FM [19] and ChemBERTA-2 [20]. PLM models typically utilize large corpus containing tens of millions of ncRNA sequences or SMILES strings for training, effectively learning substantial latent information. This information proves particularly valuable for biomedical tasks with limited annotated data. However, the application of PLMs to construct multiple modalities for MDR and MDS association prediction remains unexplored.

In this study, we propose a deep learning framework based on PLMs named PLMF-MDA, for the accurate prediction of miRNA-mediated drug resistance and sensitivity. PLMF-MDA leverages PLMs to extract global embeddings of miRNAs and drugs, and combines multi-scale convolutional neural network (CNN) and GCN to capture finer-grained embeddings at the nucleotide and atom levels, respectively. Furthermore, a cross-modal attention fusion module is incorporated to maximize the integration of node embeddings from different modalities. Our main contributions are summarized as follows:

- We present PLMF-MDA, a novel cross-modal fusion framework based on PLMs, which aims to fuse multimodal features of miRNAs and drugs to enhance the prediction performance of miRNA-drug resistance and sensitivity associations.
- We achieve comprehensive extraction of global and intrinsic embedding for miRNAs and drugs by integrating advanced PLMs like RNA-FM and ChemBERTa-2, multi-scale CNN, and GCN. The cross-modal attention mechanism adaptively fuses these embeddings, facilitating robust representation learning.
- Extensive experiments on two manually curated benchmark datasets demonstrate that PLMF-MDA consistently outperforms existing methods by leveraging both PLM and intrinsic embeddings to boost prediction performance. Further case studies substantiate the model’s effectiveness in discovering novel drug resistance and sensitivity-related miRNAs.

## Materials and methods

### Benchmark datasets

**Fig 1:**
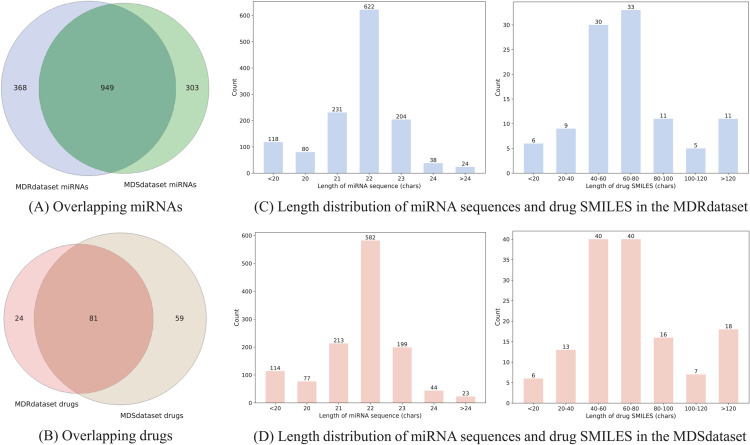
Data distribution in the benchmark datasets.

Due to the limited availability of miRNA-drug resistance and miRNA-drug sensitivity data, we manually constructed two benchmark datasets, MDRdataset and MDSdataset, based on the latest ncRNADrug database [21]. This database, published in 2023, focuses on collecting manually curated and computationally predicted drug resistance/sensitivity-related ncRNAs (miRNAs, lncRNAs, circRNAs). In this study, we primarily collected experimentally validated human miRNA-mediated drug resistance and sensitivity data. Furthermore, miRNAs that had been removed from the miRBase v22 [22] and non-small molecule drugs were excluded. Finally, after screening and preprocessing, we obtained 5411 resistance associations between 1317 miRNAs and 105 drugs as well as 5054 sensitivity associations between 1252 miRNAs and 140 drugs. Additionally, miRNA sequence information and drug SMILES were downloaded from miRBase v22 and DrugBank [23], respectively. Statistical analysis revealed that 949 miRNAs were potentially associated with both drug resistance and sensitivity during disease treatment, and 81 drugs appeared in both datasets (as shown in Fig 1A and 1B. As illustrated in Fig 1C and 1D, for the constructed MDRdataset and MDSdataset datasets, 98% of miRNA sequences were shorter than 24 nucleotides, and over 80% of drug SMILES strings had a length of 100 characters. Therefore, the maximum lengths of miRNA sequences and drug SMILES strings were fixed at 24 and 100, respectively. The basic statistics of the two datasets are presented in Table 1.

**Table 1:** The statistics of the benchmark datasets.

Datasets	miRNAs	drugs	Associations
MDRdataset	1317	105	5411
MDSdataset	1252	140	5054

### Overview

**Fig 2:**
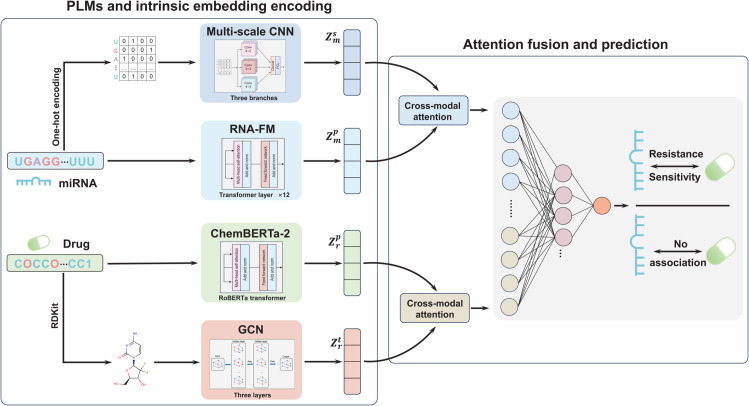
Overview of PLMF-MDA framework.

In this section, we present a novel framework PLMF-MDA for predicting miRNAs-drug resistance and sensitivity associations (see Fig 2). Our approach leverages PLMs and intrinsic embedding extractors to comprehensively encode both sequences and molecular structures. By integrating multi-perspective representations through a cross-modal attention fusion mechanism, the framework jointly learns informative embeddings from both global and fine-grained modal. Through supervised training, it effectively captures the interactions between miRNAs and drugs, thereby improving prediction accuracy.

### PLM embedding extractors for miRNA and drug

#### PLM for miRNA.

RNA-FM is a universal RNA language model built on a 12-layer bidirectional transformer encoder and trained through self-supervised learning on 23 million ncRNA sequences from the RNAcentral database [19]. This self-supervised learning approach is based on the BERT language model architecture. Through this process, RNA-FM enhances its understanding of sequence distributions and patterns related to potential structural and functional information. It has been demonstrated that embeddings generated by RNA-FM consistently outperform state-of-the-art methods in various downstream prediction tasks related to structure and function. In this work, given a miRNA sequence *S^m^* of length *L*, RNA-FM generates high-dimensional representations containing global sequence features. Specifically, the model produces an *L* × 640 embedding matrix $Z_{m}^{plm}\in {ℛ}^{L\times 640}$, where each position in the sequence is represented by a 640-dimensional feature vector.

$$Z_{m}^{plm}=RNA-FM\left(S^{m}\right)$$ (1)

Subsequently, we utilize [CLS] token, resulting in a 640-dimensional embedding vector that summarizes the entire sequence. This vector is then passed through a fully connected neural network (FCNN) projection layer to adapt the general feature space to the requirements of specific downstream tasks:

$$Z_{m}^{p}={FCNN}_{M}\left(Z_{m}^{plm}\right)$$ (2)

where $Z_{m}^{p}\in {ℛ}^{1\times dm}$ denotes the miRNA embedding obtained via PLM, and *dm* represents the final miRNA embedding dimension.

#### PLM for drug.

ChemBERTA-2 is a chemical language model based on the RoBERTa transformer implementation from HuggingFace [20]. This model was trained on data from PubChem, containing up to 77 million compound molecules. ChemBERTA-2 utilizes a masked language modeling (MLM) approach, in which 15% of the tokens in each input string are masked and the model is trained to correctly predict these masked tokens. In this study, given a drug SMILES sequence *S^r^*, we selected ChemBERTA-2 to encode it and generate drug molecular embeddings $Z_{r}^{plm}\in {ℛ}^{L\times 384}$. Given the drug SMILES, features $Z_{r}^{plm}$ are derived from the PLM:

$$Z_{r}^{plm}=ChemBERTa\left(S^{r}\right)$$ (3)

For each drug of length *L* input to ChemBERTA, the model produces an embedding matrix of size L×384. Similarly, we extract the first [CLS] token to obtain a one-dimensional vector of size 384, which is refined through FCNN projection layer to further adapt the drug embedding for downstream tasks:

$$Z_{r}^{p}={FCNN}_{R}\left(Z_{r}^{plm}\right)$$ (4)

where $Z_{r}^{p}\in {ℛ}^{1\times dr}$ denotes the drug embedding obtained via PLM, and *dr* represents the final drug embedding dimension.

### Intrinsic embedding extractors for miRNA and drug

#### Sequence embedding extractor for miRNA.

To comprehensively extract task-specific features from miRNA sequences with varying lengths and motif complexities, we designed a multi-scale CNN feature extractor. This architecture consists of multiple parallel convolutional layers with different kernel sizes, each layer for capturing motifs at different spatial resolutions. Specifically, each input miRNA sequence is first one-hot encoded to obtain a two-dimensional matrix of size *L* × 4, where *L* represents the miRNA sequence length and 4 represents the size of the base symbol dictionary. As mentioned previously, 98% of miRNA sequences in this dataset are shorter than 24 nt. To facilitate the training process, sequences are adjusted to a uniform length of 24 through truncation or zero-padding. The standardized sequences are then fed into a trainable embedding layer, which maps each nucleotide to a dense miRNA embedding matrix *M*. Next, the embedding *M* are processed in parallel by the multi-scale CNN layers. The multi-scale CNN primarily consists of three parallel convolutional branches with kernel sizes of 2, 3, and 4, respectively. Each branch is responsible for extracting features corresponding to dinucleotide, trinucleotide, and tetranucleotide sequence patterns. For each branch, the sequence embedding is first processed by a 1D convolutional layer, followed by nonlinear activation and max pooling. These steps are formally described as follows:

$$Z_{m}^{CNN}=concat\left(CNN\left(W^{\left(k\right)},M,k\right)\right),k=2,3,4$$ (5)

where *k* represents the convolutional kernel size, *W^(k)^* represents the weight matrix for the branch with kernel size *k*, and concat denotes concatenating the outputs of all branches to form a comprehensive multi-scale feature representation of the miRNA sequence. Finally, this concatenated vector is passed through a FCNN layer with dropout to obtain the final sequence encoding $Z_{m}^{s}\in {ℛ}^{1\times dm}$. This multi-scale CNN framework enables the model to capture rich and diverse sequence patterns, effectively encoding local information in miRNA sequences.

#### Graph feature extractor for drug.

For the drug feature extractor, to effectively utilize the connectivity properties between drug atoms, we convert SMILES to undirected molecular graphs using RDKit. A drug molecular graph can be represented as G=(ν,ε), where each node *a*_*i*_ represents an atom in the compound, and each edge $e_{i,j}\in \varepsilon$ represents a chemical bond between atoms *a*_*i*_ and *a*_*j*_. The initial node features *X* of the molecular graph are constructed based on the chemical properties of each atom, including atom type, hybridization type, atomic degree, formal charge, number of hydrogen atoms, number of radical electrons, etc. The drug encoder takes the molecular graph *G* as input and learns *d*-dimensional representations for each atom. In this paper, we employ GCN as the drug encoder, which is a powerful variant of graph neural networks that has been widely used as a feature extractor for various graph data. Specifically, GCN first collects feature vectors of all atoms in the neighborhood, performs aggregation operations to obtain “messages”, which are then used to update each atom’s features. Formally, given a drug graph G=(ν,ε), GCN takes its adjacency matrix *A* and node features *X* as input. We employ a three-layer GCN, with each layer can be represented as follows:

$$Z_{r}^{\left(l+1\right)}=\sigma \left(\hat{A}X^{\left(l\right)}W^{\left(l\right)}\right)$$ (6)

where $\hat{A}={\hat{D}}^{-\frac{1}{2}}(A+I){\hat{D}}^{-\frac{1}{2}}$ represents the normalized adjacency matrix, *I* is the identity matrix, and $\hat{D}$ is the degree matrix of *A* + *I*. *X^(l)^* represents the drug embedding matrix of the *l*-th hidden layer, with *X*^(0)^ = *X*. *W^(l)^* is the weight matrix of the *l*-th GCN layer, and *σ* is the ReLU function. To obtain the overall drug representation, various aggregator architectures can be applied to aggregate node (atom) representations, such as mean or max pooling. In this work, we combine both mean and max pooling by summing their outputs, resulting in the final molecular graph representation $Z_{r}^{t}\in {ℛ}^{1\times dr}$, thereby better preserving the high-level node representation.

### Cross-modal attention fusion network

Cross-attention has shown outstanding performance in multimodal fusion and feature interaction tasks by guiding information interaction between distinct modalities [24]. In this work, we obtain two types of miRNA embedding, derived from PLM encoder (RNA-FM) that captures global contextual semantics $Z_{m}^{p}$, and produced by a multi-scale CNN encoder that captures local motif patterns $Z_{m}^{s}$. Similarly, for drugs, we generate drug embedding $Z_{r}^{p}$ and $Z_{r}^{t}$ from ChemBERTA-based transformer encoder representing chemical language features and GCN reflecting molecular topological structure, respectively. To effectively integrate miRNA and drug feature obtained from PLMs and Intrinsic embedding extractors, we propose a fusion module based on cross-modal attention.

Taking the fusion of miRNA embedding $Z_{m}^{p}$ and $Z_{m}^{s}$ as an example, the following computations are performed. First, query matrices are generated based on miRNA PLM embedding $Z_{m}^{p}$, then key and value matrices are derived from the miRNA sequence embedding $Z_{m}^{s}$:

$$Q_{m}^{\left(h\right)}=Z_{m}^{p}W_{Q}^{\left(h\right)}$$ (7)

$$K_{m}^{\left(h\right)}=Z_{m}^{s}W_{K}^{\left(h\right)}$$ (8)

$$V_{m}^{\left(h\right)}=Z_{m}^{s}W_{K}^{\left(h\right)}$$ (9)

where $W_{Q}^{\left(h\right)}$, $W_{K}^{\left(h\right)}$, $W_{K}^{\left(h\right)}$ are learnable parameter matrices, and h=1,…,H corresponds to attention heads. Then, the attention score for a single head is calculated as:

$${Atten}_{m}^{\left(h\right)}\left(Z_{m}^{p},Z_{m}^{s}\right)=softmax\left(\frac{Q_{m}^{\left(h\right)}{\left(K_{m}^{\left(h\right)}\right)}^{T}}{\sqrt{d_{k}}}\right)V_{m}^{\left(h\right)}$$ (10)

where “$(\cdot {)}^{T}$” denotes matrix transpose, and *d*_*k*_ = *dm*/*H*. The outputs from all attention heads are concatenated to produce the updated miRNA features:

$${MHAtten}_{m}\left(Z_{m}^{p},Z_{m}^{s}\right)=concat\left({Atten}_{m}^{\left(1\right)}\ldots ,{Atten}_{m}^{\left(H\right)}\right)W^{\left(o\right)}$$ (11)

where *W*^(*o*)^ is a learnable parameter matrix, and *concat* represents the concatenation operation. Furthermore, we employ a residual connection and normalization to preserve original feature information.

$$Z_{m}^{ps}=LayerNorm\left(Z_{m}^{p}+{MHAtten}_{m}\right)$$ (12)

To ensure bidirectional information flow, we also compute *MHAtten*_*m*_ $\left(Z_{m}^{s},Z_{m}^{p}\right)$ in parallel to obtain updated miRNA features $Z_{m}^{sp}$. The two fusion outputs are then selectively aggregated:

$$Z_{m}=Z_{m}^{ps}+Z_{m}^{sp}$$ (13)

where *Z*_*m*_ represents the final miRNA vector that fuses global contextual semantics and local motif patterns. Similarly, given each drug’s ChemBERTA-based global features $Z_{r}^{p}$ and GCN-based topological features $Z_{r}^{t}$, cross-attention is used to obtain the final drug vector *Z*_*r*_. By adopting cross-attention fusion at the intra-modal level, our framework effectively leverages the advantages of both PLMs and deep learning extractors, thereby providing more robust and informative representations for MDR and MDS association prediction.

### Optimization objective and classification

Utilizing the fused embeddings produced by the cross-modal attention module, we constructed a feed-forward predictor to estimate the likelihood of interaction between a given miRNA and drug pair. The predictor is implemented as a FCNN that receives the concatenated miRNA and drug fusion embeddings as input and outputs a probability score. Formally, given the embedding *Z*_*m*,*i*_ and *Z*_*r*,*j*_ for the *i*-th miRNA and *j*-th drug, the prediction process is defined as:

$${\hat{y}}_{ij}=FCNN\left(Z_{m,i}\|Z_{r,j}\right)$$ (14)

where “‖” denotes vector concatenation, and FCNN(·) consists of a fully connected layer with activation function ReLU and dropout. The output ${\hat{y}}_{ij}$ represents the predicted association probability of association between the miRNA and drug. The model is trained using binary cross-entropy loss:

$$ℒ=-\frac{1}{T}\sum_{(i,j)\in T}y_{ij}\log\left({\hat{y}}_{ij}\right)+\left(1-y_{ij}\right)\log\left(1-{\hat{y}}_{ij}\right)$$ (15)

where *y*_*ij*_ represents the true association (resistance or sensitivity) between the *i*-th miRNA and *j*-th drug, and *T* is the training set of MDR (MDS) pairs.

## Experiments and results

### Experimental setup

We implemented the proposed PLMF-MDA framework in Python using the popular deep learning library PyTorch and trained the model with the Adam optimizer. Both the learning rate and weight decay were set to 5e-4. The implementation relies on several key libraries, including PyTorch v2.1.2, PyTorch-Geometric v2.4.0, RDKit v2023.9.6, and NumPy v1.24.4. All experiments were conducted on a system equipped with an RTX 4070 laptop GPU to accelerate model training and inference. The final miRNA (drug) embedding dimension *dm* (*dr*) was set to 128. For sequence feature extraction, convolutional kernels of sizes 2, 3, and 4 were used, while molecular graph features were extracted using a three-layer GCN. Additional implementation details are available on GitHub: https://github.com/sheng-n/PLMF-MDA.

To evaluate PLMF-MDA, we focus on two key metrics: area under the receiver operating characteristic curve (AUC) and area under the precision-recall curve (AUPR). These metrics are particularly suited for assessing binary classification tasks, which are prevalent in biomolecular interaction prediction. AUC measures the model’s ability to distinguish between classes, while AUPR is especially informative for imbalanced datasets.

### Performance comparison on benchmark datasets

We assessed the prediction performance of PLMF-MDA using 5-fold cross-validation (5-cv) on two manually curated benchmark datasets, MDRdataset and MDSdataset. In our experiments, known MDR (MDS) associations served as positive samples, while unknown associations were considered candidate negatives. To ensure balanced sample sizes, we randomly selected negative samples to match the number of positive samples from the negative pool. Both positive and negative samples were then randomly split into 5 folds. In each round, 4 folds were used for training and the remaining fold for testing. This procedure was repeated five times, with each fold serving as the test set once. PLMF-MDA was benchmarked against several baseline methods, including GCNNMMA [25], SubMDTA [26], GraphDTA [27], and ML_DTI [28]. All models were evaluated using 5-cv under identical conditions. Given that miRNA sequences are considerably shorter than protein sequences, we fine-tuned the competing methods to better suit the miRNA-drug association prediction task and optimize their performance.

GCNNMMA is a drug-miRNA association prediction model that employs GNN to learn molecular structural features of drugs and CNN to capture sequence features of miRNAs.

SubMDTA is a drug-target affinity prediction model that uses graph isomorphism network and Bi-directional LSTM to encode drug structural features and protein sequence features. In this study, protein sequences were replaced with miRNA sequences to enable the prediction of MDR and MDS associations.

GraphDTA is a GNN-based drug-target binding affinity prediction model. It utilizes multiple GNN architectures and CNN to extract drug structural and protein sequence representations, respectively.

ML-DTI is a sequence-based drug-target interaction prediction model that combines CNN encoder with mutual learning to learn and refine sequence features from drugs and targets.

**Fig 3:**
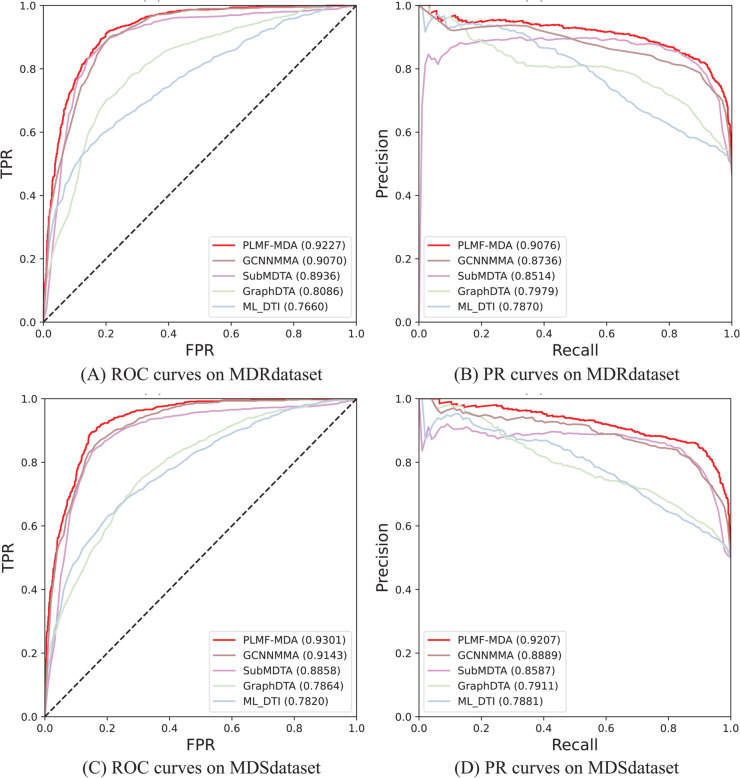
Overall performance of PLMF-MDA and baseline methods on benchmark datasets.

All models were evaluated using 5-cv under identical conditions. Given that miRNA sequences are considerably shorter than protein sequences, we fine-tuned the competing methods to better suit the miRNA-drug association prediction task and optimize their performance. The experimental results for PLMF-MDA and baseline methods are summarized in Fig 3, leading to the following key observations and analysis: (1) PLMF-MDA consistently outperformed all baseline methods on both benchmark datasets, achieving AUC and AUPR of 0.9222 and 0.9062 for the MDR association prediction task, and 0.9301 and 0.9207 for the MDS association prediction task. (2) PLMF-MDA demonstrated a substantial performance advantage over GCNMMA, SubMDTA, GraphDTA, and ML-DTI. This superiority can be attributed to PLMF-MDA’s multi-source information fusion strategy, which substantially enhances model performance compared to approaches utilizing only drug molecular graph and miRNA sequences. In summary, we hypothesize that the strong performance of PLMF-MDA is due to the integration of PLM and intrinsic embeddings for miRNAs and drugs. Next, we further verify this hypothesis through experiments in subsequent sections.

### Contribution of the PLMs and intrinsic embeddings

PLMs generate node embeddings that capturing global semantic information to characterizes molecules. In contrast, multi-scale CNN and GCN focus on extracting task-specific intrinsic features. Combining these two types of representations not only enhances the performance of existing networks but also improves the model’s generalizability in predicting miRNA-drug associations involving unseen miRNAs and drugs.

**Fig 4:**
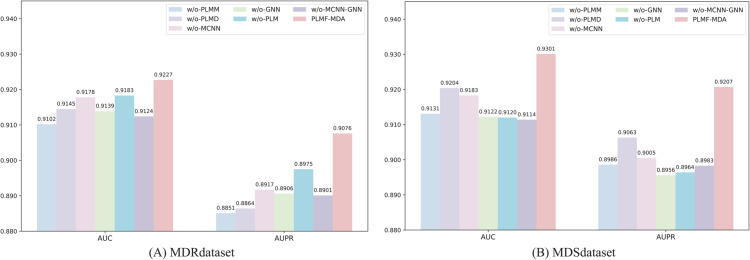
Performance of PLMF-MDA and variants.

To assess the contributions of PLMs and intrinsic embeddings, we designed six PLMF-MDA variants: (1) PLMF-MDA (w/o-PLMM), which omits the PLM-based miRNA embedding extractor; (2) PLMF-MDA (w/o-PLMD), which omits the PLM-based drug embedding extractor; (3) PLMF-MDA (w/o-MCNN), which omits the multi-CNN miRNA sequence embedding extractor; (4) PLMF-MDA (w/o-GCN), which omits the GCN drug graph embedding extractor; (5) PLMF-MDA (w/o-PLM), which omits both PLM-based miRNA and drug embedding extractors; and (6) PLMF-MDA (w/o-MCNN-GNN), which omits both multi-CNN and GNN intrinsic embedding extractors. These ablation models were evaluated using 5-cv on the benchmark MDRdataset and MDSdataset, with detailed performance results presented in Fig 4. The results show that PLMF-MDA consistently achieves higher AUC and AUPR values compared to the six ablation variants, indicating that both the PLMs and intrinsic feature extractors offer advantages for miRNA and drug representation. Importantly, although the removal of any single embedding feature leads to a performance decrease, the overall reduction is modest. This suggests that the features exhibit some degree of information overlap or correlation, allowing the model to maintain high prediction accuracy even when partial information is lost, thereby demonstrating the robustness of the proposed architecture.

### Performance of “orphan” drugs and miRNAs

**Fig 5:**
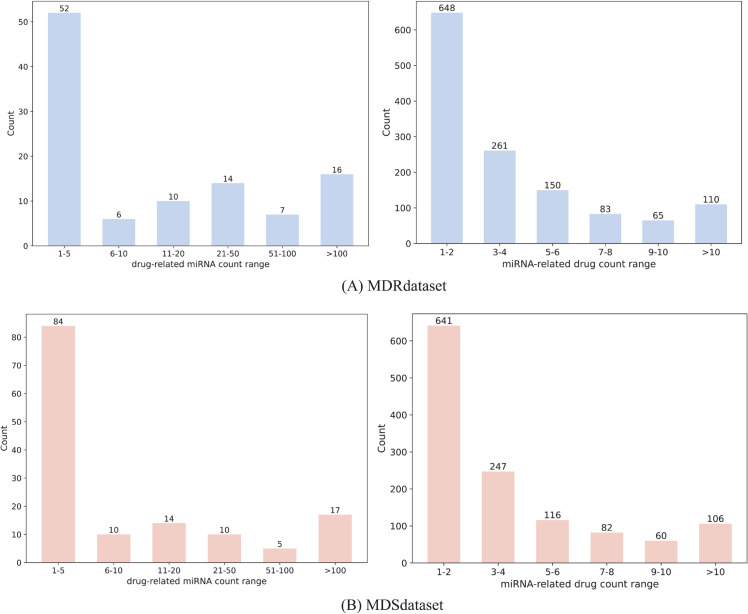
Distribution of drug (miRNA)-associated miRNA (drug) numbers in the MDRdataset and MDSdataset.

To further evaluate PLMF-MDA under realistic and challenging conditions, we tested the model in cold-start (unknown-node) scenarios. These scenarios simulate practical situations where a model must generalize to new drugs or miRNAs whose associations were not observed during training. We considered two cold-start settings: (1) drug cold, test drugs are absent from the training set. (2) miRNA cold: test miRNAs are absent from the training set. Here, known drug (miRNA) associated to miRNAs (drugs) in the benchmark dataset exhibit a long-tail distribution (as shown in Fig 5). To obtain stable and meaningful evaluation results while still reflecting realistic scarcity, we selected test nodes from intermediate frequency ranges rather than randomly. For the drug cold experiments, we selected drugs that are associated with 11-20 miRNAs. For the miRNA cold experiments, we selected miRNAs that are associated with 5-6 drugs.

The experimental results are presented in Table 2, which yields several observations: (1) The full PLMF-MDA consistently outperforms most ablated variants in both cold-start settings, demonstrating that PLMs and intrinsic embedding supply complementary information that increases robustness to unseen nodes. (2) The drug cold is substantially more challenging than miRNA cold. This may be because the dataset covers only a limited set of drug categories, which insufficient to fully characterize chemical diversity, while encompassing most human miRNAs. (3) Removing either molecular graph features or PLM-based drug embeddings substantially degrades drug cold performance (e.g., removing molecular graph features reduces MDR AUC from 0.6642 to 0.5833). This indicates that both graph topology and PLM-derived global features are essential for generalizing to novel drugs. (4) Under miRNA cold-start settings, PLMF-MDA remains highly robust, exhibiting only modest performance degradation. This suggests that RNA-level PLMs combined with multi-scale CNNs capture generalizable sequence signals that transfer well to unseen miRNAs.

**Table 2:** Performance of PLMF-MDA under orphan drugs and miRNAs.

	Drug cold				miRNA cold			
	MDR		MDS		MDR		MDS	
	AUC	AUPR	AUC	AUPR	AUC	AUPR	AUC	AUPR
w/o-PLMM	0.6498	0.6343	0.5666	0.5741	0.9008	0.8851	0.9079	0.8901
w/o-MCNN	0.6019	0.6166	0.6092	0.5865	0.9055	0.8836	0.9066	0.8860
w/o-PLMD	0.6392	0.6355	0.6244	0.6198	0.9089	0.8860	0.9055	0.8868
w/o-GCN	0.5833	0.6072	0.5883	0.5544	0.9047	0.8825	0.9067	0.8871
w/o-PLM	0.5821	0.5822	0.5723	0.5821	0.9031	0.8862	0.9064	0.8826
w/o-MCNN-GNN	0.5968	0.5728	0.5978	0.5790	0.9028	0.8817	0.9018	0.8803
PLMF-MDA	**0.6642**	**0.6534**	**0.6579**	**0.6352**	**0.9115**	**0.8977**	**0.9190**	**0.9037**

### Analysis of main parameters

**Fig 6:**
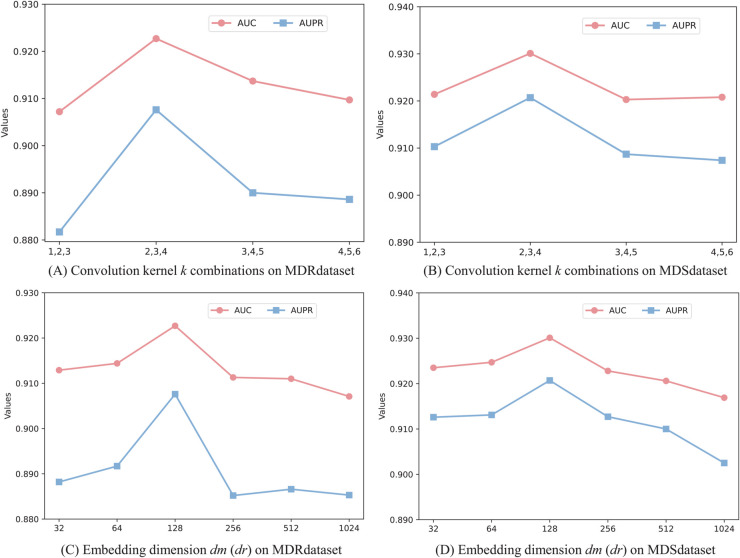
Sensitivity analysis of main hyperparameters.

In this section, we systematically investigated the sensitivity of two key parameters: the convolution kernel *k* combinations in multi-scale CNNs and the miRNA (drug) embedding dimension *dm* (*dr*). We conducted 5-cv experiments on the MDRdataset and MDSdataset datasets and reported AUC and AUPR values. **Analysis of *k***. Convolutional kernels of varying scales determine the combination pattern of *k* nucleotides. Given the short length of miRNA sequences, this study selected only four distinct kernel combinations: (1,2,3), (2,3,4), (3,4,5), and (4,5,6). The experimental results are shown in Fig 6A and 6B. The combination of convolutional kernels has a certain impact on model performance. On both datasets, the combination (2,3,4) achieved the highest AUC and AUPR. **Analysis of *dm* (*dr*)**. Next, we tested the effect of the embedding dimension of miRNA and drug, varying it from 32 to 1024. The results are shown in Fig 6C and 6D. It can be observed that performance initially improve with the embedding dimension before gradually declining. On one hand, lower embedding dimensions may be insufficient to capture complex biomolecular features; on the other hand, excessively high dimensions may lead to model overfitting and increase computational complexity. Ultimately, an embedding dimension of 128 was selected for both MDR and MDS association prediction tasks.

### Identification of resistance and sensitivity-related miRNAs for docetaxel and gefitinib

Docetaxel (ID: DB01248) is a clinically effective antimitotic agent widely used in the treatment of various cancers, including breast cancer, ovarian cancer, and non-small cell lung cancer. Previous research has shown that docetaxel resistance and sensitivity are closely linked to a variety of human miRNAs [29]. To thoroughly validate the ability of PLMF-MDA to uncover novel MDR and MDS associations, we first performed a focused case study on docetaxel. In this analysis, all known miRNAs associated with docetaxel resistance or sensitivity were masked as unknown, thereby considering docetaxel as a novel drug. The training set consisted of all remaining known MDR and MDS associations in the dataset. After model training, PLMF-MDA generated resistance and sensitivity scores for each candidate miRNA in relation to docetaxel. The top 10 miRNAs were then identified based on their predicted scores in descending order, as presented in Table 3. Our findings reveal that among the top 10 miRNAs predicted to be associated with docetaxel resistance and sensitivity, 8 candidates were corroborated by databases. This result highlights the good prediction capability of PLMF-MDA in identifying potential miRNA-drug associations.

**Table 3:** The top-10 predicted docetaxel resistance and sensitivity-associated miRNAs by PLMF-MDA.

Rank	Resistance miRNAs	Evidences	Rank	Sensitivity miRNAs	Evidence
1	hsa-miR-17-5p	ncRNADrug	1	hsa-miR-34a-5p	ncRNADrug
2	hsa-miR-181a-5p	ncRNADrug	2	hsa-miR-100-5p	ncRNADrug
3	hsa-miR-106a-5p	Unconfirmed	3	hsa-miR-200c-3p	ncRNADrug
4	hsa-miR-146b-5p	ncRNADrug	4	hsa-miR-145-5p	ncRNADrug
5	hsa-miR-138-5p	ncRNADrug	5	hsa-miR-181a-5p	ncRNADrug
6	hsa-miR-21-5p	ncRNADrug	6	hsa-miR-125b-5p	ncRNADrug
7	hsa-miR-181b-5p	ncRNADrug	7	hsa-miR-129-5p	ncRNADrug
8	hsa-miR-1915-3p	Unconfirmed	8	hsa-miR-20b-5p	ncRNADrug
9	hsa-miR-100-5p	ncRNADrug	9	hsa-let-7b-5p	Unconfirmed
10	hsa-miR-146a-5p	ncRNADrug	10	hsa-miR-9-3p	Unconfirmed

Gefitinib (ID: DB00317) is a first-line therapy for non-small cell lung cancer. However, its clinical efficacy is frequently limited by the development of drug resistance. Research indicates that miRNAs may participate in gefitinib resistance and sensitivity mechanisms in cancer through multiple pathways. Accordingly, we applied PLMF-MDA to predict miRNAs associated with gefitinib resistance and sensitivity using the same evaluation strategy. As showed in Table 4, 7 of the top 10 predicted miRNAs were validated by existing databases. Overall, these results highlight the good prediction capability of PLMF-MDA in identifying potential MDR and MDS associations.

**Table 4:** The top-10 predicted gefitinib resistance and sensitivity-associated miRNAs by PLMF-MDA.

Rank	Resistance miRNAs	Evidences	Rank	Sensitivity miRNAs	Evidence
1	hsa-miR-17-5p	ncRNADrug	1	hsa-miR-126-3p	ncRNADrug
2	hsa-miR-21-5p	ncRNADrug	2	hsa-miR-200c-3p	ncRNADrug
3	hsa-miR-106a-5p	ncRNADrug	3	hsa-miR-34a-5p	ncRNADrug
4	hsa-miR-181a-5p	ncRNADrug	4	hsa-miR-630	Unconfirmed
5	hsa-miR-27a-3p	ncRNADrug	5	hsa-miR-206	ncRNADrug
6	hsa-miR-100-5p	Unconfirmed	6	hsa-let-7b-5p	ncRNADrug
7	hsa-miR-126-3p	Unconfirmed	7	hsa-miR-574-5p	Unconfirmed
8	hsa-miR-27b-3p	Unconfirmed	8	hsa-miR-16-5p	Unconfirmed
9	hsa-miR-20b-5p	ncRNADrug	9	hsa-miR-98-5p	ncRNADrug
10	hsa-miR-29c-3p	ncRNADrug	10	hsa-let-7f-5p	ncRNADrug

## Discussion and conclusion

As disease patterns evolve and the demand for precision medicine continues to grow, drug resistance has emerged as a significant challenge, profoundly affecting disease treatment and public health. Recent research has revealed that human miRNAs are closely related to drug resistance and sensitivity, making the accurate identification of MDR and MDS associations a critical step toward the advancement of personalized medicine. Leveraging the vast capacity of PLMs addresses the inherent limitations of finite biological datasets, enabling more robust and comprehensive prediction modeling. The PLM leveraging vast data, effectively addresses the limitations of biological data. In this study, we introduced PLMF-MDA, a novel multimodal framework for predicting MDR and MDS associations that harnesses the power of advanced PLMs. PLMF-MDA utilizes the RNA language model RNA-FM and the molecular language model ChemBERTa-2 to enhance global embedding extraction from biomolecules. More fine-grained nucleotide- and atom-level modal embeddings are captured by task-specific multi-scale CNN and GCN. A cross-modal attention fusion module effectively integrates the diverse modalities relevant to miRNA-drug association prediction. Comprehensive evaluation on two manually curated benchmark datasets demonstrated that PLMF-MDA consistently achieves superior AUC and AUPR scores compared to baseline methods, maintaining high prediction accuracy even on datasets containing previously unseen nodes. Ablation studies further confirmed the individual and combined contributions of the PLM and intrinsic embedding modules. Additionally, a case study involving the anticancer drug docetaxel and gefitinib showcases the framework’s potential for discovering novel MDR and MDS associations.

Despite PLMF-MDA’s advantages in predicting miRNA-drug resistance and sensitivity associations, there are still certain limitations. First, the current model lacks interpretability, future work will focus on integrating biologically meaningful features, such as conserved miRNA motifs and drug substructures, to enhance model transparency. Second, the current model primarily incorporates miRNA sequences, drug SMILES, and molecular structural information, future extensions may include additional molecular entities, such as genes and proteins, to further enrich prediction capabilities. In summary, PLMF-MDA represents a promising approach for predicting miRNA-drug resistance and sensitivity associations, but requires further refinement and optimization to achieve broader applicability and long-term sustainability in drug discovery.
